# A22 IMPACT OF COVID-19 PANDEMIC ON FOREIGN BODY INGESTION IN CHILDREN AND ADOLESCENTS: A CROSS-SECTIONAL STUDY

**DOI:** 10.1093/jcag/gwab049.021

**Published:** 2022-02-21

**Authors:** Y Zhao, L Dehbidi Assadzadeh, A Gallant, S Gorenko-Lévêque, A Chekkal, B Djoukam Mbuko, N Pierre, M Dirks, V Groleau, A Lapointe, D Ngwanou, N Piché, C Deslandres, J Gravel, P Jantchou

**Affiliations:** 1 Metabolic and Cardiovascular Health Axis, Centre Hospitalier Universitaire Sainte-Justine, Montreal, QC, Canada; 2 Service de gastro-entérologie, CHU Sainte-Justine, Montréal, QC, Canada; 3 Pediatrics, Sainte Justine University Hospital, Montreal, QC, Canada; 4 Universite de Montreal Faculte de Medecine, Montreal, QC, Canada; 5 Universite de Sherbrooke Faculte de Medecine et des Sciences de la Sante, Sherbrooke, QC, Canada; 6 Universite de Montreal Faculte de Pharmacie, Montreal, QC, Canada

## Abstract

**Background:**

Foreign Body Ingestions (FBI), sometimes associated with severe complications, are a common reason for emergency unit visits in children. In Quebec, since March 2020, the restrictions in response to the COVID-19 pandemic have increased the time children spend at home. We hypothesized that this could contribute to a rise in FBI incidence and severity.

**Aims:**

The primary objective of our study was to evaluate the incidence as well as the clinical presentation of FBI cases seen at CHU Sainte-Justine Children’s Hospital in Montreal (CHUSJ) during the COVID-19 pandemic as compared to the two previous years. Our secondary objectives were to estimate the rate of severe FBI (involving hospitalisations and/or complications) and to evaluate the nature of the foreign bodies that were ingested.

**Methods:**

All children referred to or who presented at CHUSJ for FBI between March 2018 and February 2020 (pre-pandemic) as well as between March 2020 and February 2021 (pandemic) were included (n=690). Cases of food impaction were excluded (n=78). Incidence of FBI was calculated by dividing the number of FBI cases by the total number of emergency department visits per period. Differences between the two groups were analyzed by Student T test or Chi-square test.

**Results:**

Between March 2018 and February 2021, 612 patients (median age 3.5 years (1.6–5.9); 54% male) were eligible. The mean monthly number of FBI cases (min-max) in 2020–2021 was 18.6 (9–28), significantly higher than the year 2018 [16.6 (8–22)] and the year 2019 [15.5 (9–24)]; p=0.04. The incidence rate of FBI doubled during the pandemic as compared to the prepandemic group: respectively 57.5/10,000 emergency department visits and 23.2/10,000 visits (p=0.002). Almost one fourth of the cohort was hospitalized. The hospitalization rate (>1 day) was similar between the 2 periods: 8.8% before the pandemic and 7.1% during the pandemic. Digestive endoscopy was performed in 21.5% of cases, a rate similar before and during the pandemic. A total of 3.3% of the children developed complications related to FBI. This rate remained stable between the two periods. The most frequently ingested objects were coins (25.0%), toys (10.8%), button batteries (10.6%), magnets (6.2%), and jewellery (6.2%). There was no significant difference in the nature of FB ingested between the 2 periods although the number of magnet ingestions increased during the pandemic (18 per year vs 10 per year).

**Conclusions:**

The incidence of FBI increased significantly during the pandemic in comparison with the two previous years. The high hospitalization and complications rates, although stable during the pandemic, underline the significant impact of pediatric FBI.

**Funding Agencies:**

None

